# Visualization of an Accessory Pathway by 3D High-Density Mapping: A Case of Ebstein Anomaly With Atrioventricular Re-entrant Tachycardia

**DOI:** 10.1016/j.cjco.2021.01.008

**Published:** 2021-01-21

**Authors:** Chihiro Ota, Miyako Igarashi, Akihiko Nogami, Tomoko Ishizu, Kazutaka Aonuma, Masaki Ieda

**Affiliations:** Department of Cardiology, Faculty of Medicine, University of Tsukuba, Tennodai, Tsukuba, Ibaraki, Japan

## Abstract

Radiofrequency catheter ablation (RFCA) is the primary choice for treating patients with an accessory pathway and atrioventricular re-entrant tachycardia. However, using RFCA to treat a right-sided accessory pathway in a patient with Ebstein anomaly can be difficult owing to challenges in locating the electrophysiological atrioventricular groove. We report a case of atrioventricular re-entrant tachycardia in a patient with Ebstein anomaly and a right-sided accessory pathway that was successfully treated using RFCA and 3-dimensional (3D) high-density mapping. RFCA and 3D mapping may be useful in the management of such cases and may aid in improving prognoses of patients.

## Résumé

L’ablation par radiofréquence (ARF) est le premier choix de traitement chez les patients qui ont une voie accessoire et une tachycardie atrioventriculaire par réentrée. Toutefois, l’utilisation de l’ARF pour traiter une voie accessoire du côté droit d’un patient qui a une anomalie d’Ebstein peut être difficile en raison des défis de la loca-lisation du sillon atrioventriculaire à l’exploration électrophysiologique. Nous rapportons un cas de tachycardie atrioventriculaire par réentrée chez un patient qui a une anomalie d’Ebstein et une voie accessoire du côté droit, dont le traitement au moyen de l’ARF et de la cartographie tridimensionnelle (3D) haute définition a connu une réussite. L’ARF et la cartographie 3D peuvent être utiles dans la prise en charge de ces cas et aider à améliorer le pronostic des patients.

## Case Report

A 52-year-old woman presented to our clinic with regular tachycardia of 140 beats per minute, as revealed by a portable electrocardiogram (ECG). Although she had been diagnosed with Ebstein anomaly as a child, she did not undergo corrective surgery owing to the absence of any symptoms of heart failure, cyanosis, worsening exercise capacity, and progressive right-ventricular (RV) dilatation.^1^ A 12-lead ECG performed during sinus rhythm revealed a right bundle branch block with a normal PR interval and no evidence of ventricular pre-excitation. Echocardiography showed severe right-atrial (RA) dilatation and severe tricuspid regurgitation caused by apical displacement of the posterior and medial leaflets. Left-ventricular systolic function was normal.

An electrophysiological study was performed, and orthodromic atrioventricular re-entrant tachycardia (AVRT) was repeatedly induced because of a right-sided accessory pathway. The CARTOSOUND system, intracardiac echo facilitating full mapping integration of the CARTO3 3D electroanatomic mapping system (version 6.0, Biosense Webster, Inc, Diamond Bar, CA), was used for anatomic mapping. Apical displacement of the posterior and medial leaflets of the tricuspid valve was apparent. Using this information, the site of the functional tricuspid valve annulus, which was defined as the area surrounded by the sites of attachment of the leaflets of the tricuspid valve, was manually tagged.

**Figure 1:**
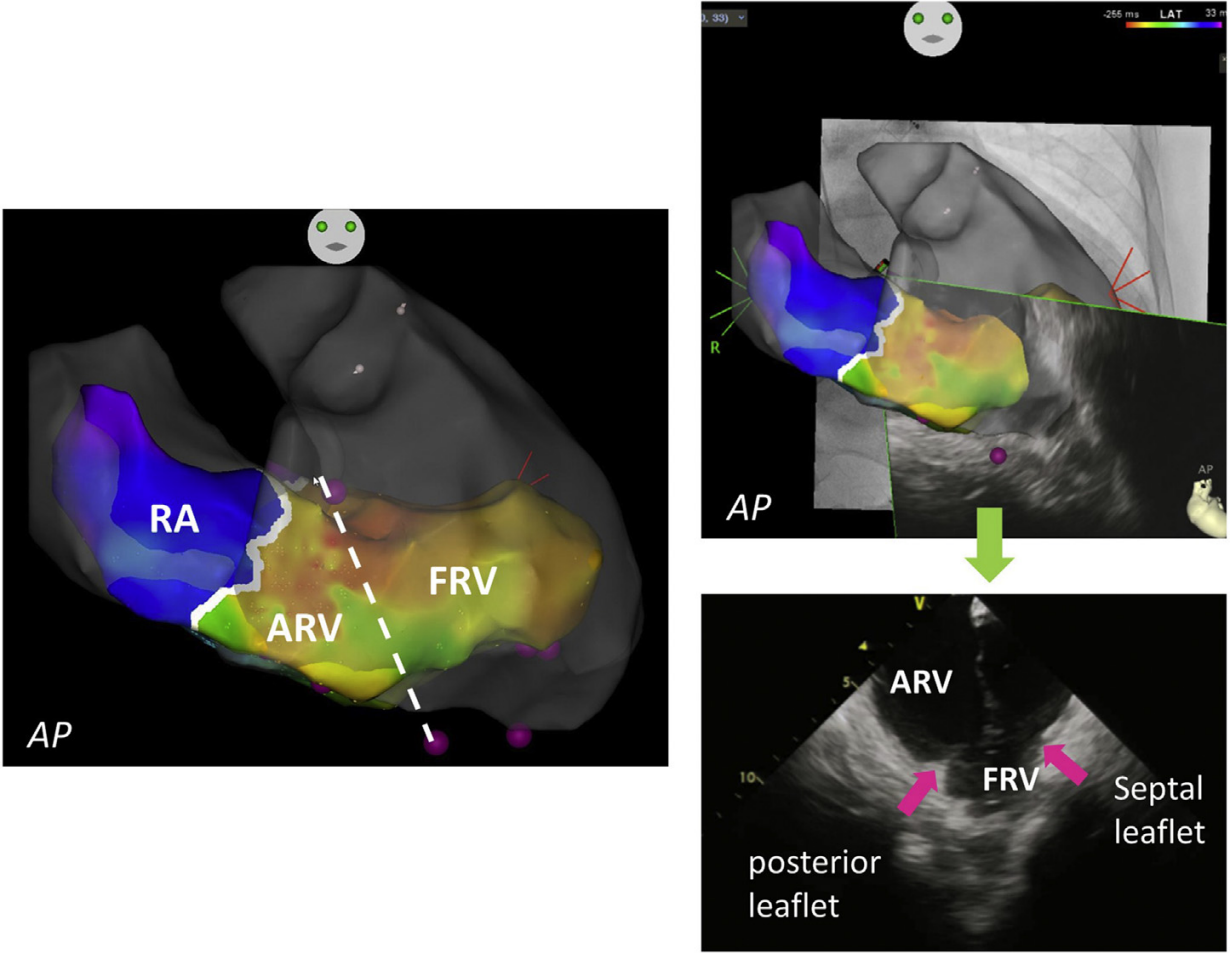
An anatomic map by CARTOSOUND (Biosense Webster, Inc, Diamond Bar, CA). The edge of the tricuspid valve leaflet is clearly observed (**pink tags** in left figure, **pink arrows** in right figure). An activation map is merged with the anatomical map. The gap between the identified true tricuspid valve annulus (**white solid line**) and the functional tricuspid valve annulus (**white broken line**) represents the ARV. ARV, atrialized right ventricle; FRV, functional right ventricle; RA, right atrium.

Subsequently, a high-density activation map of both the RA and the RV was generated, using a PentaRay catheter (Biosense Webster, Inc.) during AVRT. This system allows for the visualization of the time phase difference with a lower threshold of total activation time as potential conduction block areas.^2^ Therefore, the activation map revealed the electrical atrioventricular (AV) groove (true tricuspid valve annulus, solid white line in Fig. 1) and an overt gap between the true tricuspid valve and the functional tricuspid valve annulus (atrialized right ventricle [ARV]) (Fig. 1; Video 1 , view video online). In this patient, although both atrial and ventricular electrograms were recorded at the true tricuspid valve, the single ventricular electrogram was recorded at the functional tricuspid valve annulus. Therefore, the functional tricuspid valve was in the RV and was not the “true tricuspid valve.”

**Figure 2:**
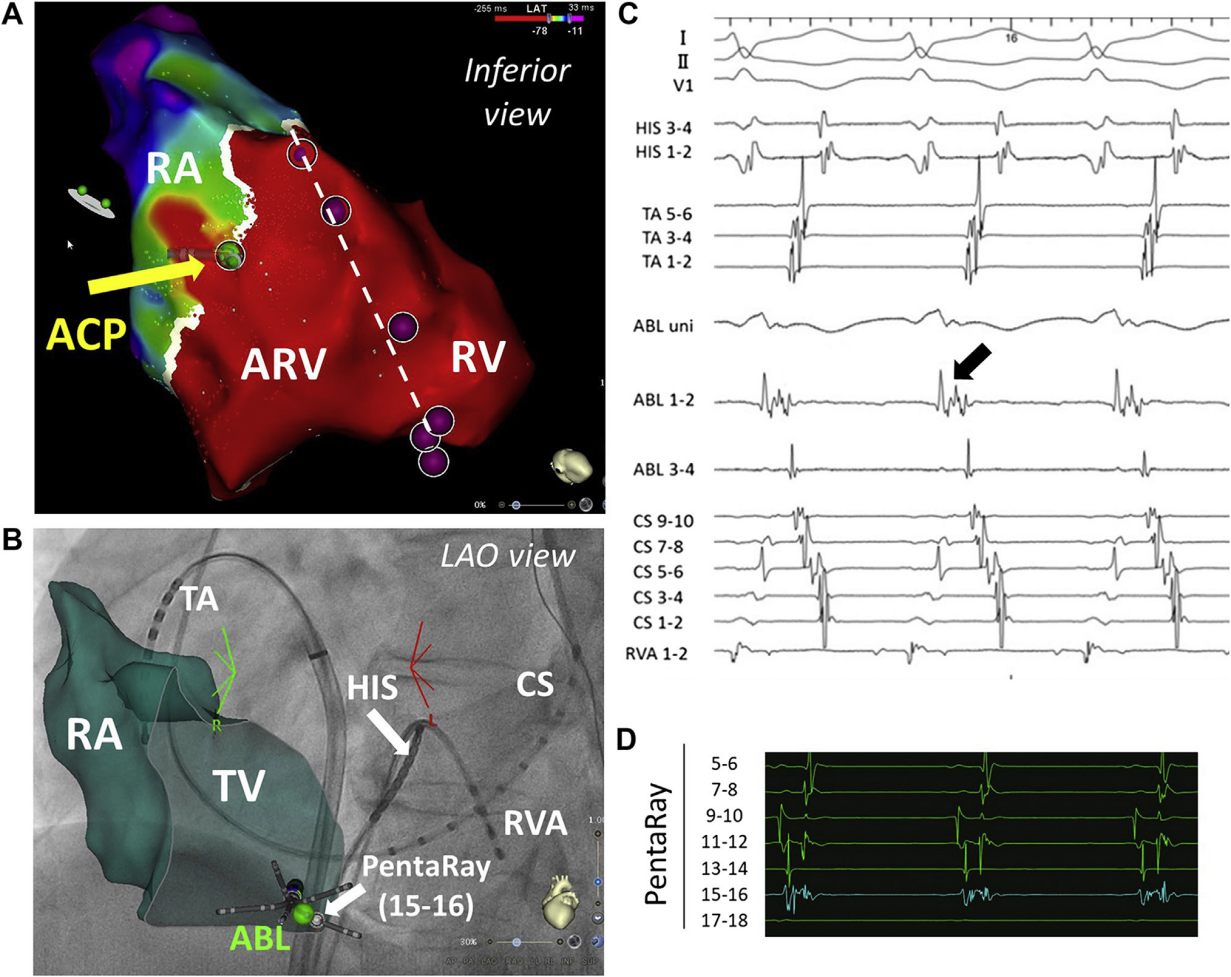
(**A**) A high-density activation map image taken during the atrioventricular re-entrant tachycardia. An obvious gap is observed between the true tricuspid valve annulus (**white lines**) and the functional tricuspid valve annulus (**white broken line, pink tags**). The accessory pathway is visualized as a segment of continuous ventriculoatrial (VA) conduction (**yellow arrow**) on the true tricuspid valve annulus. (**B**) X-ray fluoroscopy imaging of the catheter’s position was merged with a 3D mapping system. The success site was posterior to the true tricuspid valve annulus (**green tag**). (**C**) The ablation catheter at the accessory pathway shows continuous VA potentials (**black arrow**). (**D**) The local electrogram at the accessory pathway was recorded using a PentaRay catheter (Biosense Webster, Inc, Diamond Bar, CA). ABL, ablation catheter; ACP, accessary pathway; ARV, atrialized right ventricle; CS, coronary sinus; RA, right atrium; RV, right ventricle; RVA, right ventricular apex; TA, tricuspid annulus, TV, tricuspid valve.

Furthermore, an accessory pathway was apparently located at the posterior aspect of the true tricuspid valve (Fig. 2; Videos 2 and 3 , view videos online). An ablation catheter (ThermoCool SmartTouch SF; Biosense Webster, Inc) that was passed through a steerable sheath (AgilisTM NxT; Abbott Medical, Inc, Minneapolis, MN) and placed at the visualized accessory pathway showed continuous ventruculoatrial (VA) potentials. The first radiofrequency application with an output of 30 W was successful in terminating the AVRT and eliminating conduction across the accessory pathway, 2.8 seconds after initiation (average temperature: 27°C, average contact force: 6 g) (Fig. 2).

## Discussion

Ebstein anomaly is generally a malformation of the tricuspid valve with myopathy of the RV that has variable presentations of anatomic and pathophysiological characteristics.^3^ The major abnormality in Ebstein anomaly is a characteristically enlarged and fenestrated anterior tricuspid valve leaflet.^3^ In most patients, the posterior and septal leaflets are hypoplastic and apically displaced and tend to adhere to the RV. These abnormalities of the tricuspid valve result in a reduction in the size of the functioning RV, depending on the severity of its atrialization. Moreover, tricuspid regurgitation leads to dilatation of the atrialized RV and RA.^3^^,^^4^

Arrhythmias are common in patients with Ebstein anomaly. Previous reports have indicated that atrial flutter, atrial tachycardia, and AVRT caused by accessory pathways are related to Ebstein anomaly.^5^

Class I indications for surgical repair in patients with Ebstein anomaly include heart failure symptoms, cyanosis, progressive RV dilatation, and worsening exercise capacity,^1^ which were absent in our patient. She only had palpitation caused by AVRT; hence, she was a candidate for surgical repair with a class IIa indication, as are all patients with tachyarrhythmia. However, according to recent evidence, RFCA is the first choice of treatment for tachyarrhythmia, even in patients with Ebstein anomaly.

Although RFCA is generally the first choice for treating an accessory pathway in patients with Wolff-Parkinson-White syndrome, its success rate is lower in patients with Ebstein anomaly than in those with structurally normal hearts.^6^ One possible reason for this is the possibility of identifying multiple accessory pathways.^7^ Another reason is difficulty in identifying the true tricuspid valve annulus.

In the current case, anatomic mapping of the RA and RV using the CARTOSOUND system was performed before RFCA. This allowed the apically displaced posterior and septal leaflets to be clearly recorded. The area surrounded by the attachment sites of the leaflets of the tricuspid valve was defined as the functional tricuspid valve annulus. Subsequently, activation mapping was performed during AVRT using CARTO3 version 6.0. In this mapping, the true tricuspid valve annulus was revealed as a possible conduction block (white lines), whereas the accessory pathway was visualized as a part with continuous VA conduction (the gap between the white solid lines).

High-density activation mapping of both the atrium and ventricle combined with CARTOSOUND is useful in all cases in which RFCA is used to treat an accessory pathway. Accessory pathways in the free wall are especially susceptible to mechanical pressure. If its conduction is transiently blocked by mechanical pressure during mapping, using an ablation catheter, it becomes impossible to map and ablate the accessory pathway. Therefore, activation mapping using a PentaRay catheter is comparatively better when avoiding mechanical bumps, as the tip of the catheter is softer than that of an ablation catheter.

However, we believe that 3D mapping is best suited for treating a right-sided accessory pathway in patients with Ebstein anomaly because conventional RFCA procedures already have a high rate of success in the treatment of accessory pathways in structurally normal hearts.

Another problem encountered during RFCA of an accessory pathway in patients with Ebstein anomaly is the difficulty in catheter contact and stability at the free wall of the tricuspid valve annulus. A steerable sheath is useful in improving contact and stability during positioning even in a patient with an enlarged RA and RV. In our case, Agilis NxT (Abbott Medical, Inc., Minneapolis, MN) was used as the ablation catheter, and this may have contributed to a smooth and successful ablation.

**Novel Teaching Point**

• High-density activation mapping of both the atrium and ventricle combined with CARTOSOUND is useful for an accessory pathway ablation, especially in patients with altered anatomy, such as in Ebstein anomaly.

## Conclusions

By using CARTOSOUND and high-density mapping during AVRT, the functional tricuspid valve and the true tricuspid valve annulus can be visualized in a patient with Ebstein anomaly. Similarly, the accessory pathway was accurately visualized, collectively leading to the successful elimination of the accessory pathway using RFCA.

## Funding Sources

No funding sources were provided for this case report.

## Disclosures

Dr Igarashi has received endowments from Boston Scientific, Japan Lifeline, Nihon Kohden Inc, Biotronik, Toray, Abbott Medical Inc, and Astec Co, Ltd. Dr Nogami has received honoraria from Abbott Medical Inc. and Daiichi-Sankyo, and an endowment from Medtronic. All other authors have no conflicts of interest to disclose.

## Supplementary Material

Vidoe 1 **The movie with rotation of the CARTO maps, including the activation map merged with the anatomical shell, which was created using CARTOSOUND.** The pink tags represent the edge of the tricuspid valve leaflets. The white solid lines represent the true tricuspid valve annulus. The gap between the white lines and the pink tags represents the atrialized right ventricle. The green tag at the inferior part of the true tricuspid valve annulus is the site of successful ablation of the accessory pathway.

Video 2 **A high-density activation map during atrioventricular reentrant tachycardia.** The white lines represent the time phase difference with lower range of activation time, indicating a potential functional block. In this case, these lines illustrate the “true” tricuspid valve annulus. Ventriclo-atrial conduction is represented by the gap between the lines located posterior to the tricuspid valve annulus. Therefore, this area represents the accessory pathway. RA = right atrium, ARV = atrialized right ventricle, RV = right ventricle.

Video 3 **A reconstructed coherent map evaluated during atrioventricular reentrant tachycardia.** Coherent mapping was performed using CARTO 3 version 7.0 (Biosense Webster, Diamond Bar, CA, USA). This map was reconstructed at a later date because the system was not available at our institution when the radiofrequency catheter ablation was initially performed. This system improves the representations of electrical wave propagation through coloring and direction vectors. The reconstruction includes the detection of areas with slow or nonconducting (SNO) zones (brown areas). Arrows in the atrialized right ventricle indicate relayed electrical waves to the SNO zones. This represents the “true” tricuspid valve annulus. Moreover, electrical conduction broke through a single point (green tag) at the gap between the SNO zones. This point is the site of successful ablation of the accessory pathway.
