# Supplementary figures and images for: Visualization of an Accessory Pathway by 3D High-Density Mapping: A Case of Ebstein Anomaly With Atrioventricular Re-entrant Tachycardia
Source: CJC Open. 2021 Jan 21;3(6):827–30. doi: 10.1016/j.cjco.2021.01.008 (PMC8209408; doi:10.1016/j.cjco.2021.01.008)

## Slide 1
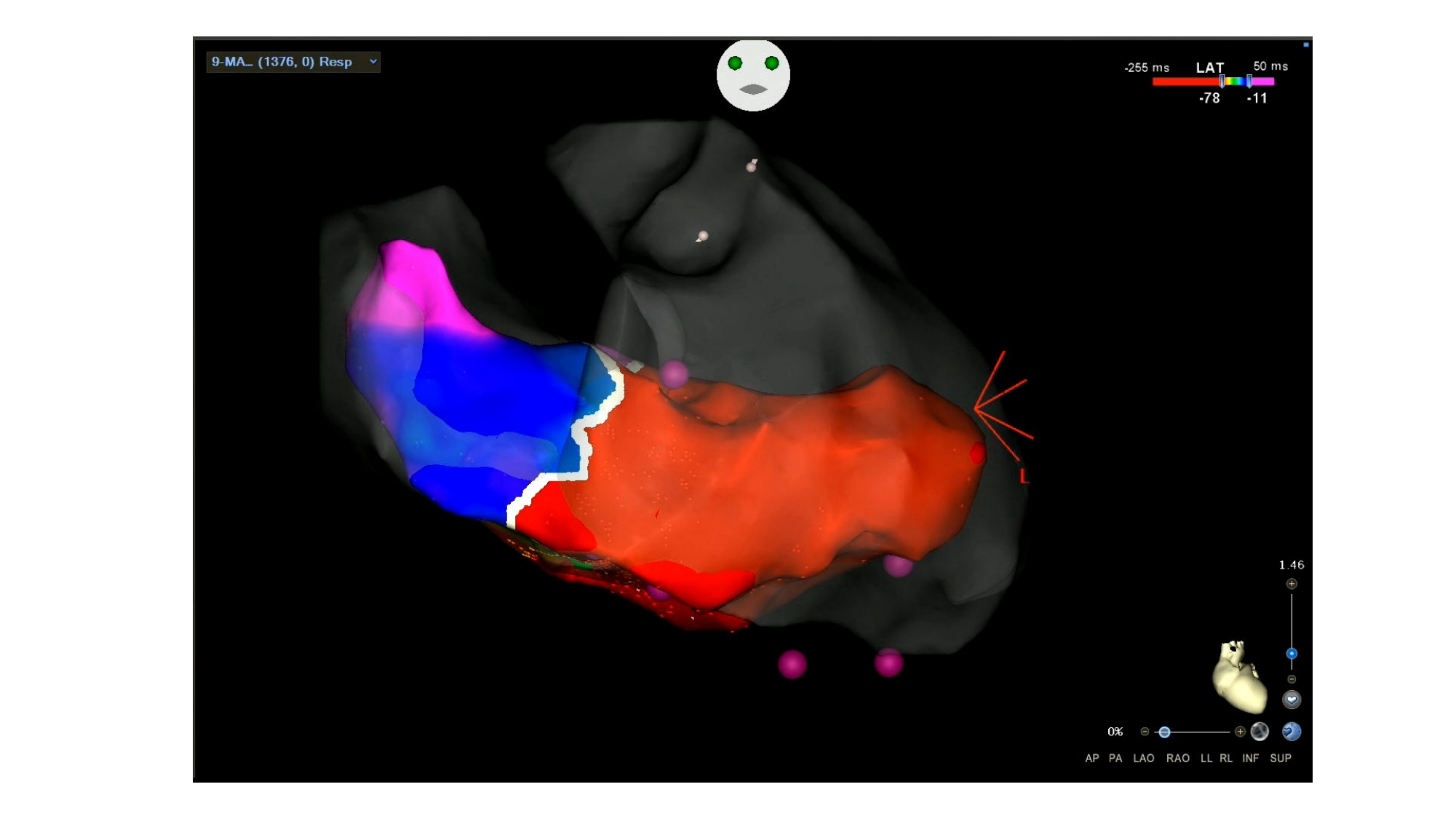

Supplement: Vidoe 1 — The movie with rotation of the CARTO maps, including the activation map merged with the anatomical shell, which was created using CARTOSOUND. The pink tags represent the edge of the tricuspid valve leaflets. The white solid lines represent the true tricuspid valve annulus. The gap between the white lines and the pink tags represents the atrialized right ventricle. The green tag at the inferior part of the true tricuspid valve annulus is the site of successful ablation of the accessory pathway. [file mmc1.pptx]

## Slide 1
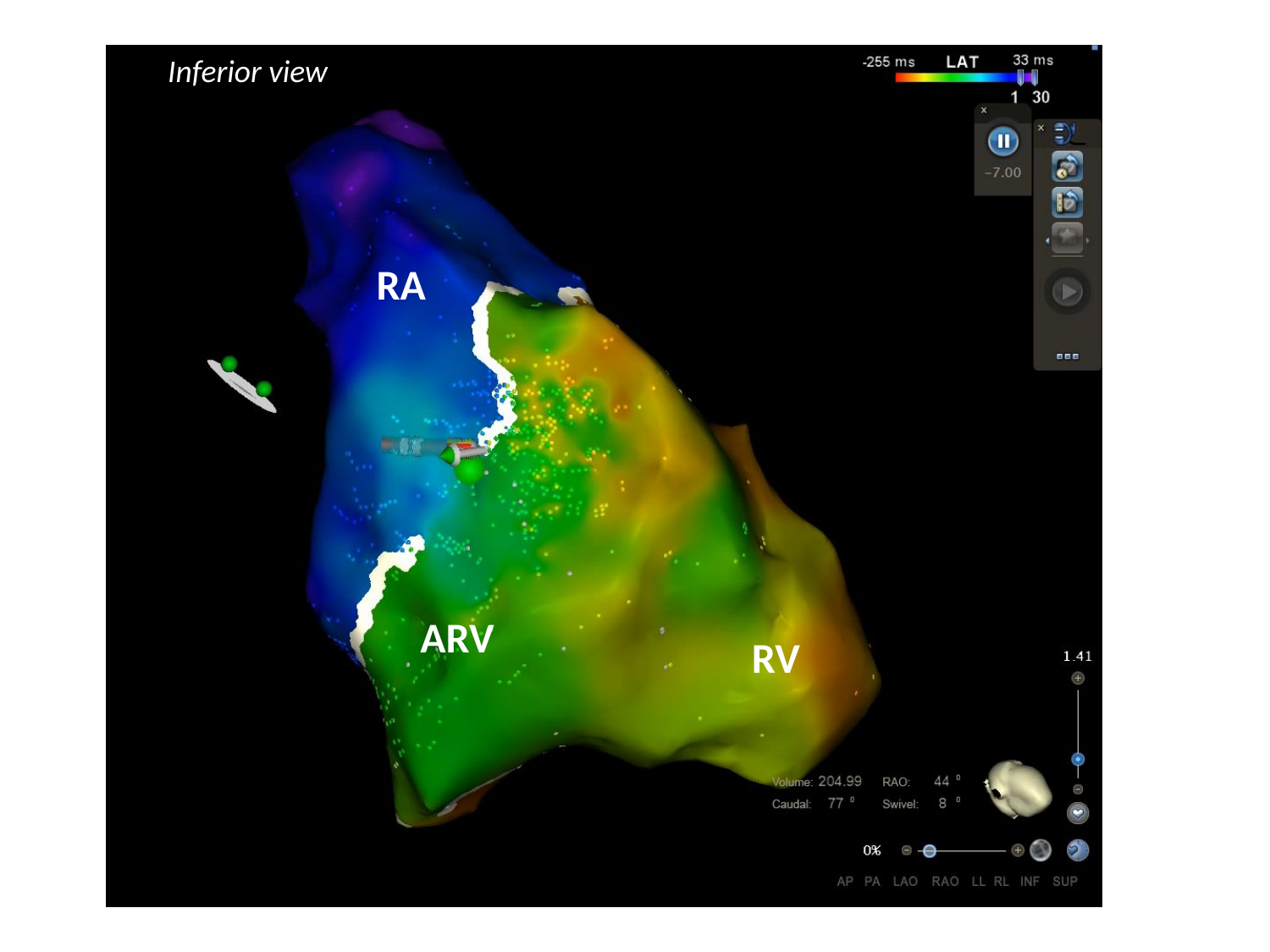

Inferior view
RA
ARV
RV

Supplement: Video 2 — A high-density activation map during atrioventricular reentrant tachycardia. The white lines represent the time phase difference with lower range of activation time, indicating a potential functional block. In this case, these lines illustrate the “true” tricuspid valve annulus. Ventriclo-atrial conduction is represented by the gap between the lines located posterior to the tricuspid valve annulus. Therefore, this area represents the accessory pathway. RA = right atrium, ARV = atrialized right ventricle, RV = right ventricle. [file mmc2.pptx]

## Slide 1
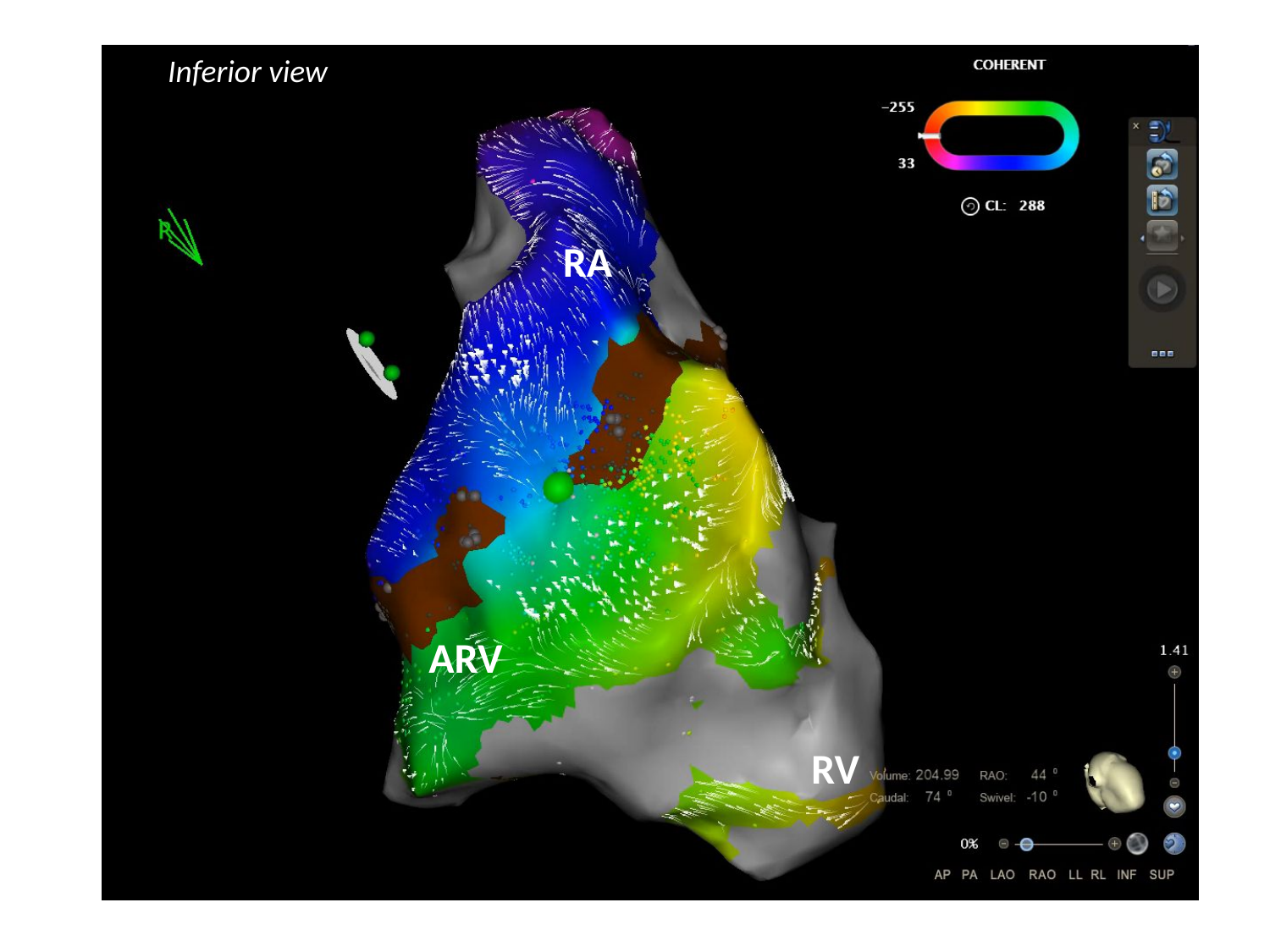

Inferior view
RA
ARV
RV

Supplement: Video 3 — A reconstructed coherent map evaluated during atrioventricular reentrant tachycardia. Coherent mapping was performed using CARTO 3 version 7.0 (Biosense Webster, Diamond Bar, CA, USA). This map was reconstructed at a later date because the system was not available at our institution when the radiofrequency catheter ablation was initially performed. This system improves the representations of electrical wave propagation through coloring and direction vectors. The reconstruction includes the detection of areas with slow or nonconducting (SNO) zones (brown areas). Arrows in the atrialized right ventricle indicate relayed electrical waves to the SNO zones. This represents the “true” tricuspid valve annulus. Moreover, electrical conduction broke through a single point (green tag) at the gap between the SNO zones. This point is the site of successful ablation of the accessory pathway. [file mmc3.pptx]
